# Application of Intestinal Barrier Molecules in the Diagnosis of Acute Cellular Rejection After Intestinal Transplantation

**DOI:** 10.3389/ti.2023.11595

**Published:** 2023-09-08

**Authors:** Yun Chen, Sheng-Hong Tseng, Chih-Yen Chen, Ya-Hui Tsai

**Affiliations:** ^1^ Department of Surgery, Far Eastern Memorial Hospital, New Taipei City, Taiwan; ^2^ Graduate Institute of Medicine, Yuan Ze University, Taoyuan, Taiwan; ^3^ Department of Surgery, National Taiwan University Hospital, Taipei, Taiwan; ^4^ Medicine and Institute of Emergency and Critical Medicine, School of Medicine, National Yang Ming Chiao Tung University, Taipei, Taiwan; ^5^ Division of Gastroenterology and Hepatology, Taipei Veterans General Hospital, Taipei, Taiwan; ^6^ Association for the Study of Small Intestinal Diseases, Taoyuan, Taiwan

**Keywords:** biomarkers, acute rejection, intestinal transplant, intestinal barrier, noninvasive

## Abstract

Diagnosing acute rejection after intestinal transplantation currently heavily relies on histopathological analysis of graft biopsies. However, the invasive risks associated with ileoscopic examination and the inaccessibility for biopsy after ileostomy closure hinder real-time detection of rejection responses. Molecules comprising the intestinal barrier have been identified as physiological and molecular biomarkers for various bowel conditions and systemic diseases. To investigate the potential of barrier function-related molecules in diagnosing rejection after intestinal transplantation, plasma samples were collected longitudinally from transplant recipients. The samples were categorized into “indeterminate for rejection (IND)” and “acute rejection (AR)” groups based on clinical diagnoses at each time point. The longitudinal association between plasma levels of these barrier function-related molecules and acute rejection was analyzed using the generalized estimating equations (GEE) method. Logistic GEE models revealed that plasma levels of claudin-3, occludin, sIgA, and zonulin were independent variables correlated with the clinical diagnosis of acute rejection. The subsequent prediction model demonstrated moderate ability in discriminating between IND and AR samples, with a sensitivity of 76.0%, specificity of 89.2%, and accuracy of 84.6%. In conclusion, monitoring plasma levels of claudin-3, occludin, sIgA, and zonulin shows great potential in aiding the diagnosis of acute rejection after intestinal transplantation.

## Graphical Abstract

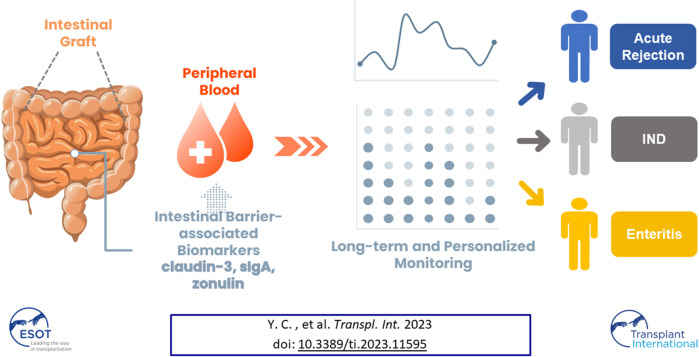

## Introduction

Intestinal transplantation (ITx) is considered the definitive treatment for patients with irreversible intestinal failure or life-threatening complications after long-term reliance on parenteral nutrition [1, 2]. The small intestine, with its abundant lymphoid tissue and diverse bacterial flora, has a higher incidence of acute rejection compared to other organ transplants [3, 4]. Approximately 50%–75% of small bowel transplantation patients experience acute rejection, ranging from mild forms with cryptic apoptosis to severe cases that result in ulcerative destruction of the epithelial mucosa, posing a challenge to graft and patient survival [3–6].

At present, the gold standard for diagnosing acute rejection following ITx depends on endoscopic observation and biopsy histology [7, 8]. However, the discontinuation of scheduled biopsies after ileostomy closure poses challenges in the early detection of acute rejection [9, 10]. Therefore, the identification of novel molecular biomarkers that can be non-invasively detected with high accuracy has been a crucial goal in aiding the clinical detection of rejection in intestinal transplantation [11–13].

The intestinal barrier plays a pivotal role in maintaining immune response homeostasis and immune tolerance by protecting the mucosal surface of the intestine [14–17]. The “microbiota-immune axis” concept has linked the intestinal barrier to various pathological conditions. Impairment of the intestinal barrier can lead to increased microbial translocation, inducing pro-inflammatory conditions in the intestine and subsequent systemic disorders [15–19]. Research has identified junctional molecules such as claudins, occludin, zonula occludens-1 (ZO-1), and regulatory proteins like secretory IgA (sIgA) and zonulin as potential biomarkers for several pathological conditions, including inflammatory bowel disease (IBD), irritable bowel syndrome (IBS), food allergy, metabolic diseases, and leaky gut syndrome [20–23].

From 2007 to 2022, we conducted 31 ITx surgeries for 30 patients, with 5 year survival rates of 71.0% for patients and 51.6% for grafts, comparable to global figures [4, 5, 24]. To improve long-term outcomes by reducing graft loss related to acute rejection, we aimed to explore non-invasive biomarkers to enhance the accuracy and timeliness of acute rejection diagnosis. This study aimed to investigate the correlation between molecular levels of intestinal barrier components in plasma and the incidence of acute rejection, with the goal of developing a predictive model for diagnosing acute rejection.

## Materials and Methods

### Study Design and Sample Collection

To establish a time-series database for monitoring the plasma levels of intestinal barrier molecules in intestinal transplant recipients, plasma sample collection commenced on the day of transplantation, prior to the operation. Subsequent plasma collections followed the blood draw schedule outlined in the post-transplant monitoring protocol (see below). Blood samples were promptly transferred into heparin-containing tubes upon collection. After undergoing standardized centrifugation at 300×g, the plasma was divided into polypropylene tubes and stored at −80°C until analysis. The listing of plasma samples was documented based on the clinical manifestations and diagnosis recorded on each respective day.

### Post-Transplant Monitoring Protocol and Diagnosis of Acute Rejection

At the time of ITx surgery, a Santulli’s proximal chimney ileostomy was created in each recipient for endoscopic examination and biopsy of the graft. The frequency of endoscopic examination was twice a week in the first month, once a week in the second month, once every other week in the third month, once a month in the fourth to sixth month, and whenever necessary.

The frequency of drawing blood was per day in the first week, twice a week in the second to the fourth week, once a week in the second month, once every other week in the third month, once a month in the fourth to sixth month, and whenever necessary.

The diagnosis of acute rejection was established through the pathological analysis of the biopsy, in conjunction with the identification of significant morphological changes in the graft mucosa during endoscopic examination [3, 25].

### Quantification of Plasma Levels of the Intestinal Barrier Molecules

The plasma samples were thawed and vortexed before being subjected to ELISA assays. The procedure for detection and determination of their concentrations were performed according to the manufacturer’s protocols. The ELISA kits used in the study included: Citrulline (CEA505Ge, Cloud-Clone Corp., Katy, TX 77494, USA), Claudin-1 (CSB-EL005490HU, Cusabio Life Science, Houston, TX 77054, USA), Claudin-2 (CSB-EL005500HU, Cusabio Life Science), claudin-3 (CSB-EL005505HU, Cusabio Life Science), Claudin-4 (CSB-E17961h, Cusabio Life Science), L-FABP (HA404-1, Hycult Biotech Inc., Wayne, PA 19087, USA), Occludin (SEC228Hu, Cloud-Clone Corp.), sIgA (SEA641Hu, Cloud-Clone Corp.), zonular occludens-1 (CSB-E13916h, Cusabio Life Science) and zonulin (K5601, Immundiagnostik AG, 64625 Bensheim, Germany).

### Statistical Analysis

This study employed generalized estimating equations (GEE) models to account for the effect of repeated measures, with Patient ID serving as the subject variable to define individual subjects within the dataset. Age and the concentrations of ten barrier function-related molecules were treated as continuous variables, while gender was considered as a categorical variable. The biopsy result was used as the binary outcome variable. Binary logistic GEE analysis was utilized to calculate the regression coefficients and odds ratios for the independent variables. The predictive probability of acute rejection and the clinical incidence of acute rejection were further analyzed using ROC (Receiver Operating Characteristic) curves.

Statistical analysis was conducted using SPSS software (version 22.0, IBM Corp., Chicago, IL, USA). The statistical data are presented as mean ± SE. The significance level was indicated by *p*-values, with a value of *p* < 0.05 considered statistically significant for all analyses.

## Results

### Patients and the Grouping of Plasma Samples

A total of 172 time-series plasma samples were collected from seven patients between September 2016 and June 2022, along with their corresponding medical records, including histopathological reports of graft biopsies during the same period. Plasma samples corresponding to non-rejection intestinal conditions (e.g., enteritis) and other systemic situations (e.g., sepsis) were excluded from the analysis. The basic information of the seven patients and the number of plasma samples collected are presented in Table 1. Next, based on clinical findings and/or biopsy reports on the day of blood collection, 143 plasma samples were categorized as IND (indeterminate for acute rejection, *n* = 93) and AR (acute rejection, *n* = 50). The mean plasma levels of ten intestinal barrier-related molecules are shown in Table 2.

**TABLE 1:** Basic characteristics of the patients whose plasma samples were used in this research.

Patient ID	Age	Gender	No. of plasma samples	Episode(s) of AR	Severity and timing of AR^a^
Pt-1	31	Female	42	4	mild (D13)
					severe (D30)
					severe (D175)
					severe (D234)
Pt-2	58	Male	21	2	mild (D21)
					mild (D82)
Pt-3	29	Female	22	0	
Pt-4	37	Male	23	2	severe (D16)
					mild (D73)
Pt-5	58	Female	14	1	severe (D36)
Pt-6	28	Female	10	1	mild (D20)
Pt-7	63	Male	11	0	

^a^ Timing of AR was represented as the day after transplant.

**TABLE 2:** Mean plasma levels of intestinal barrier molecules in the IND and AR groups.

	IND (Mean ± S.E.)	AR (Mean ± S.E.)	Unit
N	93	50	
citrulline	17.03 ± 0.50	16.21 ± 0.51	ng/mL
claudin-1	309.76 ± 65.01	240.09 ± 15.05	pg/mL
claudin-2	319.76 ± 38.20	276.58 ± 31.86	pg/mL
claudin-3	76.20 ± 5.07	109.45 ± 8.04	pg/mL
claudin-4	47.43 ± 5.29	50.62 ± 8.08	pg/mL
L-FABP	21.40 ± 2.30	16.52 ± 2.00	ng/mL
occludin	4.02 ± 0.43	2.34 ± 0.25	ng/mL
sIgA	120.17 ± 7.24	81.64 ± 5.26	μg/mL
ZO-1	403.13 ± 25.04	437.91 ± 30.32	pg/mL
zonulin	5.99 ± 0.32	4.02 ± 0.24	ng/mL

### Univariate Analysis

The association between plasma levels of intestinal barrier molecules and the diagnosis of AR was investigated by using univariate GEE analysis (Table 3). Among the examined variables, claudin-3 demonstrated a significant positive association with AR (coefficients = 0.013, *p* < 0.001). Conversely, citrulline demonstrated a significant negative association with acute rejection (coefficient = −0.121, *p* = 0.022). Notably, occludin and zonulin also exhibited significant negative association with acute rejection with the coefficients −0.339 (*p* = 0.010) and −0.367 (*p* < 0.001), respectively. The remaining variables, including claudin-1, claudin-2, claudin-4, L-FABP, sIgA, ZO-1, did not demonstrate statistically significant associations with AR in this univariate analysis (Table 3).

**TABLE 3:** Longitudinal association between the plasma levels of intestinal barrier molecules and acute rejection by univariate GEE analysis.

	Regression coefficient	Standard Error	Wald	*p*-value	OR	95% C.I. for OR	
						Lower	Upper
citrulline	−0.121	0.053	5.245	0.022	0.886	0.799	0.983
claudin-1	<0.001	<0.001	0.056	0.813	1.000	0.999	1.001
claudin-2	<0.001	0.001	0.156	0.693	1.000	0.999	1.001
claudin-3	0.013	0.003	15.078	< 0.001	1.013	1.006	1.020
claudin-4	0.003	0.003	0.814	0.367	1.003	0.997	1.009
L-FABP	<0.001	<0.001	0.840	0.360	1.000	1.000	1.000
occludin	−0.339	0.132	6.601	0.010	0.712	0.550	0.923
sIgA	−0.002	0.004	0.442	0.506	0.998	0.990	1.005
ZO-1	<0.001	0.001	0.361	0.548	1.000	0.999	1.002
zonulin	−0.367	0.082	20.113	< 0.001	0.693	0.590	0.813

### Multivariable Analysis

Further multivariate GEE analysis was conducted to better understand the collective impact of these molecules on the risk of acute rejection. In Table 4, three regression models revealed certain significant associations between plasma levels of intestinal barrier molecules and acute rejection. Claudin-3 demonstrated a consistent positive association with acute rejection across all models, with odds ratios (OR) of 1.026 (95% C.I. 1.012–1.040, *p* < 0.001) in model 1, 1.025 (95% C.I. 1.013–1.037, *p* < 0.001) in model 2, and 1.022 (95% C.I. 1.011–1.032, *p* < 0.001) in model 3. On the other hand, occludin showed consistent negative associations with acute rejection, with ORs of 0.566 (95% C.I. 0.390–0.820, *p* = 0.003) in model 1, 0.627 (95% C.I. 0.459–0.857, *p* = 0.003) in model 2, and 0.574 (95% C.I. 0.417–0.791, *p* = 0.001) in model 3. Zonulin also exhibited a significant negative association with acute rejection, with ORs of 0.743 (95% C.I. 0.582–0.947, *p* = 0.016) in model 1, 0.778 (95% C.I. 0.631–0.960, *p* = 0.019) in model 2, and 0.817 (95% C.I. 0.684–0.975, *p* = 0.025) in model 3. Additionally, sIgA demonstrated a significant negative association with acute rejection in model 1, with an OR of 0.986 (95% C.I. 0.973–0.999, *p* = 0.031). However, other variables, including citrulline, claudin-1, claudin-2, claudin-4, L-FABP, and ZO-1, did not exhibit statistically significant associations with acute rejection in the multivariable analysis models.

**TABLE 4:** Multivariate GEE analyses of the association between the plasma levels of intestinal barrier molecules and acute rejection.

	Model 1^a^		Model 2^a^		Model 3^a^	
	OR^b^ (95% C.I.)	*p*-value	OR (95% C.I.)	*p*-value	OR (95% C.I.)	*p*-value
claudin-3	1.026 (1.012–1.040)	<0.001	1.025 (1.013–1.037)	<0.001	1.022 (1.011–1.032)	<0.001
occludin	0.566 (0.390–0.82)	0.003	0.627 (0.459–0.857)	0.003	0.574 (0.417–0.791)	0.001
zonulin	0.743 (0.582–0.947)	0.016	0.778 (0.631–0.960)	0.019	0.817 (0.684–0.975)	0.025
sIgA	0.986 (0.967–0.999)	0.031	0.990 (0.978–1.001)	0.082		
citrulline	0.906 (0.791–1.039)	0.157				
claudin-1	1.000 (0.999–1.001)	0.972				
claudin-2	1.001 (0.999–1.004)	0.315				
claudin-4	1.007 (0.996–1.017)	0.209				
L-FABP	1.000 (1.000–1.000)	0.847				
ZO-1	0.999 (0.996–1.001)	0.258				
QICC^b^	150.572		139.552		145.405	
sensitivity	72.0%		76.0%		68.0%	
specificity	94.6%		89.2%		79.6%	
accuracy	86.7%		84.6%		75.5%	

^a^ Analyses were adjusted for gender and age.

^b^ OR, odds ratio; QICC, corrected quasi likelihood under independence model criterion.

### Evaluation of the AR Prediction Models

The model performance, as assessed by the QICC (corrected quasi-likelihood under the independence model criterion), showed that GEE model 2 had the lowest value (QICC = 139.552), suggesting a better fit compared to model 1 (QICC = 150.572) and model 3 (QIC = 145.405). The diagnostic sensitivity, specificity, and accuracy of model 2 were 76.0%, 89.2%, and 84.6%, respectively (Table 4).

**FIGURE 1:**
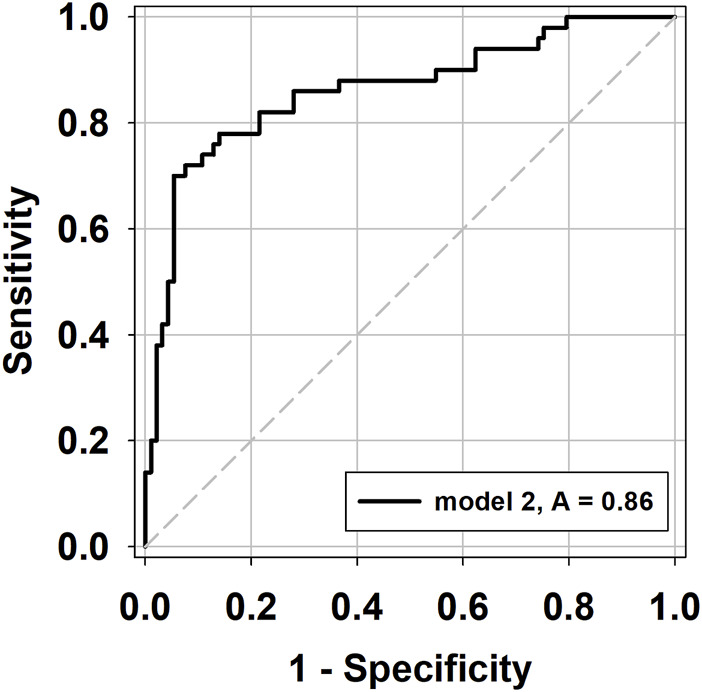
ROC curves of the predictive probability (PP) values from GEE model 2 in the prediction of acute rejection.

The predictive probability of acute rejection was calculated for each sample using regression model 2, and the relationship between the predictive probability and the incidence of acute rejection was analyzed using the ROC curve. The AUC was calculated as 0.862 (95% C.I. 0.794 to 0.930, *p* < 0.001), with a model probability cut-off of 0.432 being identified as the optimal threshold (Figure 1).

## Discussion

In the present study, our results demonstrated that there were significant changes of claudin-3, occludin, sIgA, and zonulin during the onset of acute rejection after intestinal transplantation. These four molecules were independent factors most related to the clinical diagnoses of acute rejection, with that the increase in claudin-3 was associated with higher probability of acute rejection while increased occludin, sIgA and zonulin were negatively associated with acute rejection.

Endoscopic examination and tissue biopsy, as the most conventional method for graft monitoring, is still holds as the most definite way of confirming the diagnoses of rejection after intestinal transplantation [8, 9]. The search for non-invasive biomarkers for diagnosing acute rejection had been on in the recent decade. For example, blood citrulline and stool calprotectin had been considered as potential biomarkers for this purpose. Decreased citrulline was reported to reflect reduced enterocyte mass and intestinal insufficiency during acute rejection [11, 26, 27]; increased fecal calprotectin implicated ongoing immune responses in the intestine [28]. However, the lack of diagnostic specificity had limited their application in diagnosing acute rejection [29–31].

The molecules regulating intestinal barrier function had been identified as biomarkers to evaluate intestinal permeability thus being applied in the diagnosis of inflammatory bowel diseases [20–23]. We therefore investigated the applicability of these biomarkers in the detection of acute rejection after intestinal transplantation. As the results shown, we have tracked down to four molecules with different roles in barrier functions.

Secretory IgA serves as a crucial defense effector in the intestinal barrier, playing a key role in microbial neutralization and immune exclusion It is produced by plasma cells in the epithelial lamina propria, transported across epithelial cells, and then secreted into the lumen [32, 33]. Quantifying sIgA in serum or saliva has been applied for diagnosing Crohn’s disease (CD), ulcerative colitis (UC), and mucositis, with elevated levels observed in active CD and reduced levels in UC [34–36]. In our study, we found a negative association between sIgA levels and the onset of acute rejection (Table 4), suggesting altered sIgA production or depletion during rejection. Intestinal microbial stimulation and Th1-inhibiting/Th2-stimulating cytokines play a role in balancing sIgA levels [37]. Given that Th1-inhibiting cytokines (e.g., IL-6, IFN-γ and TNF-α) are involved in acute rejection, the downregulation of sIgA could serve as an early indicator of the acute rejection-associated Th1 immune response.

Altered expression of claudins in intestinal tissue has been extensively studied in patients with various intestinal disorders. Reduced expression of claudin-1 was observed in patients with inflammatory bowel disease (IBD) and irritable bowel syndrome (IBS) [38, 39], while an increase in claudin-2 was found in the inflamed epithelium of patients with ulcerative colitis (UC) [40, 41]. The variation in claudin-3 and claudin-4 expression in IBD remains controversial, with studies reporting both reduced and increased expression [42–44]. In our study, we found a significant association between claudin-3 and acute rejection (Table 4), suggesting increased levels of claudin-3 in circulation due to intestinal tissue destruction during rejection.

The expression of occludin has shown variability in intestinal biopsies of patients with Crohn’s disease (CD) and ulcerative colitis (UC), suggesting inconsistent patterns in occludin expression within these studies [45, 46]. However, limited research has explored the use of plasma occludin as a marker for intestinal diseases. Interestingly, plasma occludin has gained attention in the context of blood-brain barrier damage, demonstrating fluctuating levels of occludin in different types of stroke [47].

Zonulin is an important regulator of barrier function that can disrupt the tight junctions between cells [48]. Previous research has highlighted the association between increased zonulin expression and various conditions, including inflammatory bowel disease (IBD), food allergy, diabetes, arthritis, liver disease, and aging [49–52]. In our study, we initially hypothesized that higher zonulin levels would contribute to the compromised intestinal integrity observed during acute rejection. However, contrary to our expectations, we found lower levels of zonulin in the acute rejection group. It is worth noting that the intestinal epithelial cells are a significant source of zonulin [53]. The reduction in zonulin levels in the acute rejection group could potentially be attributed to impaired or dysfunctional intestinal cells during the onset of acute rejection.

Our investigation into predictive factors for acute rejection, including claudin-3, occludin, sIgA, and zonulin, has illuminated distinct roles in maintaining intestinal barrier integrity. While claudin-3, occludin, and zonulin consistently emerged as significant factors associated with acute rejection in both univariate and multivariable analyses, sIgA demonstrated significance when other variables were considered. Excluding sIgA from model 2 led to reduced prediction sensitivity and accuracy in model 3, underscoring its crucial contribution.

ROC curves were generated to determine the optimal cutoff values for claudin-3, occludin, sIgA, and zonulin in predicting the occurrence of acute rejection. The analysis revealed that claudin-3 levels above 90.32 pg/mL (*p* < 0.001), occludin levels below 2.55 pg/mL (*p* = 0.185), sIgA levels below 63.37 μg/mL (*p* < 0.001), and zonulin levels below 2.95 ng/mL (*p* < 0.001) were indicative of the diagnosis of acute rejection, as depicted in Supplementary Figure S1. It is important to note, however, that the ROC analyses did not take into consideration the potential impact of repeated measurements within individual samples. Therefore, these cutoff values should not be used for clinical purposes at this time.

The quest for acute rejection-specific biomarkers is a challenging endeavor. Low specificity in differentiating acute rejection from enteritis complicates conclusive outcomes [31]. Due to the dispersed distribution of samples representing enteritis and sepsis outcomes within our patient cohort, we opted not to include these groups in our GEE analysis. However, we conducted a detailed comparison of relative changes in claudin-3, occludin, sIgA, and zonulin levels across the IND, AR, enteritis, and sepsis samples, as outlined in Supplementary Table S1. Noteworthy differences emerged among these sample groups, with both enteritis and sepsis-related samples displaying elevated concentrations of barrier markers compared to the IND group. In sepsis cases, we observed an exceptionally high mean level of claudin-3, spanning a wide range. This suggests the possibility that a simultaneous increase in these markers might indicate pathological conditions such as enteritis and sepsis. Importantly, this finding highlights that elevations in claudin-3 alone may not reliably indicate acute rejection, emphasizing the need for a more comprehensive diagnostic framework or a combination of markers.

Furthermore, our prediction model revealed a significant insight: the variation trends in sIgA and zonulin for patients with acute rejection were opposite to those observed in patients with other inflammatory or ulcerative intestinal diseases, both in existing literature and our own data. The mean values of sIgA and zonulin were relatively correlated with the severity of acute rejection with the AR-severe group displaying greater significance (*p* = 0.011 for sIgA; *p* = 0.002 for zonulin) than the AR-mild group (*p* = 0.023 for sIgA; *p* = 0.006 for zonulin) (Supplementary Table S2; Supplementary Figure S2). This finding holds significant potential when differential diagnoses must be made, providing a valuable advantage.

Our study, although illuminating, faces certain limitations, primarily due to a small number of patients and sample size. Enhancing the model’s sensitivity, specificity, and accuracy would benefit from additional laboratory data, including white blood cell counts, immunosuppressant concentrations, liver function parameters, and renal function indicators. Another limitation stems from the restricted quantity of plasma samples, limiting the exploration of potential molecules associated with barrier function. However, the innovative aspect of our study lies in our statistical approach, acknowledging the importance of individual variations. Accounting for repeated measures within each patient enables the capture of dynamic trends in diagnostic markers, creating a more comprehensive and reliable basis for detecting rejection. This approach differentiates our study from prior research, emphasizing the need to consider nuanced variations for a more accurate diagnosis.

## Conclusion

In conclusion, our study has identified claudin-3, sIgA, and zonulin as promising non-invasive biomarkers for diagnosing acute rejection in recipients of intestinal transplants. Notably, this is the pioneering investigation to employ GEE analysis for comparing plasma levels of intestinal barrier molecules in the rejection and non-rejection phases of intestinal transplant recipients. We anticipate that our model holds significant potential to enhance post-transplant monitoring of intestinal grafts, ultimately advancing patient care in this critical domain.

## Data Availability

The datasets presented in this article are not readily available to preserve individuals’ privacy under local IRB regulation. Requests to access the datasets should be directed to yahuitsi@saturn.yzu.edu.tw.

## Abbreviations

AR, acute rejection; CD, Crohn’s disease; IND, indeterminate for rejection; IBS, irritable bowel syndrome; IBD, inflammatory bowel disease; ITx, intestinal transplantation; GEE, generalized estimating equations; L-FABP, liver-type fatty acid-binding protein; OR, odds ratio; ROC, Receiver Operating Characteristic; sIgA, secretory immunoglobulin A; UC, ulcerative colitis; ZO-1, zonula occludens-1.
