# Supplementary material for: Application of Intestinal Barrier Molecules in the Diagnosis of Acute Cellular Rejection After Intestinal Transplantation
Source: Transpl Int. 2023 Sep 8;36:11595. doi: 10.3389/ti.2023.11595 (PMC10514359; doi:10.3389/ti.2023.11595)

Supplementary Material - 1

Table S1. Mean plasma levels of intestinal barrier molecules in different sample groups.

	IND (*Mean ± S.E.*)	*AR* (*Mean ± S.E.*)	*enteritis* (*Mean ± S.E.*)	*sepsis* (*Mean ± S.E.*)	*Unit*
**N**	93	50	*18*	*11*	
**claudin-3**	76.20 ± 5.07	109.45 ± 8.04	114.99 ± 18.91	779.58 ± 246.18	pg/ mL
**occludin**	4.02 ± 0.43	2.34 ± 0.25	8.00 ± 1.01	9.01 ± 0.72	ng/ mL
**sIgA**	120.17 ± 7.24	81.64 ± 5.26	269.08 ± 36.90	213.43 ± 23.07	μg/ mL
**zonulin**	5.99 ± 0.32	4.02 ± 0.24	6.42 ± 1.86	9.03 ± 1.28	ng/ mL

Table S2. Comparison of the plasma levels of intestinal barrier molecules between the IND and AR groups.

	IND (Mean ± S.E.)	*AR-mild (Mean ± S.E.)* / *AR-severe (Mean ± S.E.)*	*Post-Hoc[#] P-value*	*ANOVA P-value*
**claudin-3 (pg/ mL)**	76.20 ± 5.07	96.88 ± 15.26	*0.167*	***0.041***
		102.97 ± 7.53	***0.027***	
**occludin (ng/ mL)**	4.02 ± 0.43	3.66 ± 0.43	*0.988*	***0.032***
		1.67 ± 0.34	***0.023***	
**sIgA (µg/ mL)**	120.17 ± 7.24	83.37 ± 7.08	***0.023***	***0.003***
		79.92 ± 7.90	***0.011***	
**zonulin (ng/ mL)**	5.99 ± 0.32	4.14 ± 0.41	***0.006***	***<0.001***
		3.90 ± 0.27	***0.002***	

[#] Dunnett's post-hoc test with the IND group as the reference.

Figure S1. ROC curve analysis for determining the optimal cut-off values of (A) claudin-3, (B) occludin, (C) sIgA, and (D) zonulin in the prediction of AR

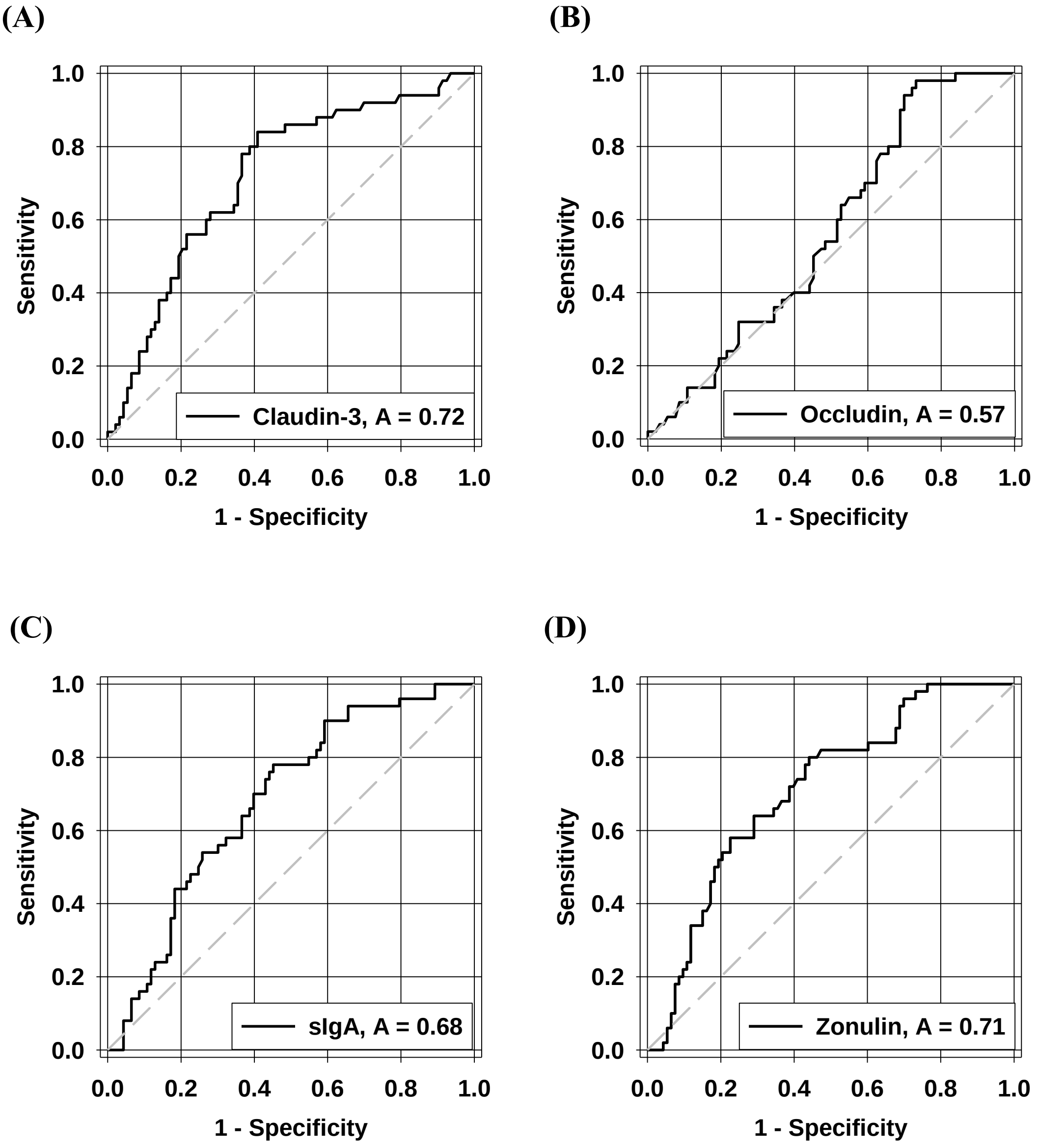

Figure S2. Boxplots of the plasma levels of claudin-3, occludin, sIgA, and zonulin in the IND, AR-mild, and AR-severe groups. The grey circle refers to outlier; the red line refers to the mean value in each group.

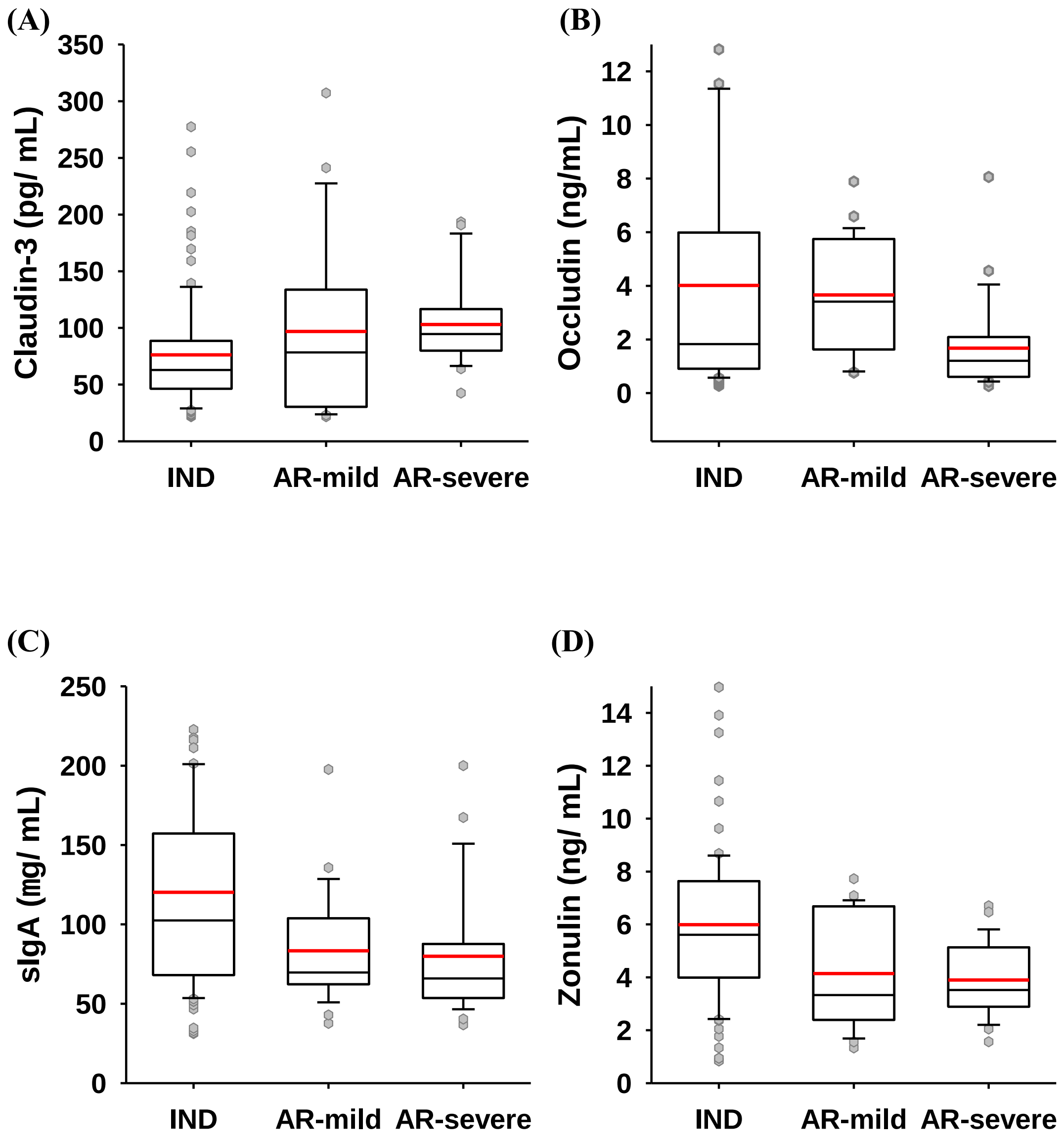

Supplement: Supplementary file 1 [file DataSheet1.pdf]
